# Antibiotic Resistance in Metal-Tolerant Microorganisms from Treatment Facilities

**DOI:** 10.3390/antibiotics12121678

**Published:** 2023-11-29

**Authors:** Leonid Perelomov, Olga Sizova, Maria Gertsen, Irina Perelomova, Vyacheslav Arlyapov, Yury Atroshchenko

**Affiliations:** 1 Laboratory of Biogeochemistry, Tula State Lev Tolstoy Pedagogical University (Tolstoy University), Lenin Avenue, 125, Tula 300026, Russia; perelomov@rambler.ru (L.P.); reaktiv@tsput.ru (Y.A.); 2 Federal Research Center “Pushchino Scientific Center for Biological Research of the Russian Academy of Sciences”, G. K. Skryabin Institute of Biochemistry and Physiology of Microorganisms of RAS, Pushchino 142290, Russia; osizova@rambler.ru; 3 Medical Institute, Tula State University, Lenin Avenue, 92, Tula 300012, Russia; ketava@rambler.ru; 4 Research Center “BioChemTech”, Tula State University, Lenin Avenue, 92, Tula 300012, Russia; v.a.arlyapov@tsu.tula.ru

**Keywords:** bacteria, resistance, antibiotics, trace elements, treatment facilities, sewage sludge

## Abstract

The study examines the antibiotic resistance of metal-tolerant bacteria isolated from the wastewater treatment plant of a large city to six antibiotics belonging to the β-lactam antibiotics, aminoglycosides and amphenicols. Resistance of bacteria from sewage sludge multitolerant to heavy metals to 18 antibiotics of the β-lactam antibiotics, tetracyclines, aminoglycosides, diaminopyrimidines, amphenicols and ansamycins was studied also. Out of 10, the metal-tolerant microorganisms isolated from wastewater treatment facilities only the *Klebsiella pneumonia* strain (tolerant to 3 mM Cu) from the sludge of a secondary settling tank did not show resistance to the studied antibiotics at the concentrations considered. Resistance to the maximum amount of antibiotics was typical for strains *Serratia fonticola* SS0-1, isolated from fresh sewage sludge and resistant to 5 mmol Cu and 3 mmol Pb, or *Stenotrophomonas maltophilia* SS0-5, also isolated from fresh sludge and resistant to 3 mmol Zn and Cu. It is possible that bacterial resistance to antibiotics develops not only as a result of the use of antibiotics themselves, but also as a result of environmental pollution with heavy metals, and vice versa.

## 1. Introduction

The use of antibiotics throughout the entire time after their discovery ensures the preservation of public health and, as a result, leads to an increase in the quality and length of life [1]. In addition to medical use, antibiotics are widely used in veterinary medicine, agriculture (livestock, poultry, fish farming, beekeeping), as well as the food industry in various technological processes [2]. The use of antibiotics worldwide is steadily growing, both due to the increase in the use of antibiotics in medicine and in other areas [3]. Thus, consumption of antibiotics in livestock exceeded 63 tons in 2010 and is predicted to increase by 67% in 2030 [4]. According to the calculations of a number of authors in some countries (China, India, Brazil, Russia, South Africa), the use of antibiotics will double by this time [4]. Excessive application of antibiotics has led to the contamination of different environments: soils, surface and groundwater and agricultural products. Persistent increased concentrations of antibiotics in the environment are the reason for the growth of antibiotic resistance in microbes that cause serious medical and environmental problems [5]. The formation of resistance of pathogenic bacteria to antibiotics can lead to treatment failure—an increase in the duration of the disease and mortality [6].

In the conditions of large cities, significant amounts of antibiotics enter the environment through the sewer network and sewage treatment facilities [7]. Sources of antibiotics for urban wastewater treatment plants can be wastewater from factories and institutions that produce and use antibiotics, as well as the excrement of residents [8]. Wastewater from pharmaceutical factories and hospitals is an important input source of antibiotic residue pollution. A high positive correlation was observed between the amount of ciprofloxacin and metronidazole used in the hospital and its content in the hospital wastewater [9]. From 25 to 90% (depending on the class of drugs) of the received dose of antibiotics is excreted in the feces and urine as an original compound—in the active form [10]. Also, some antibiotics end up in wastewater as a result of improper disposal of unused or expired medicines [11].

Trace elements are another dangerous environmental pollutant. Trace elements in high concentrations are extremely toxic to all living organisms, including humans [12]. The release of trace elements, especially heavy metals and metalloids, into the environment as a result of industrial development and urbanization is a global problem today [13]. The main amount of metals comes from industrial wastewater [14]. However, domestic and non-industrial wastewater from industrial enterprises entering municipal wastewater treatment plants may also contain significant amounts of trace elements. The sources of trace elements to domestic wastewater can be stormwater containing runoff from roofs, atmospheric depositions and products of wear of tires, building materials, pipe sediments, rest of food, or businesses using sewerage for their waste, such as, for example, car washes. Using the example of the capital of Sweden, the authors [15] showed that the largest sources of Cu were runoff from roofs and tap water. For Zn, the largest sources were galvanized material and car washes. In the case of Ni, the largest sources were chemicals used in the wastewater treatment plants and the drinking water itself. The dominant emission source of Hg was the teeth amalgam. For Cd, Pb and Cr, the largest contributors were car washes. In addition, emergency discharges of industrial enterprises into the sewer system are possible [15].

Microorganisms fiercely compete with each other, which causes the production of various antibiotic molecules that prevent the proliferation of other bacteria [16]. To ensure their survival, bacteria have successfully developed mechanisms to resist the massive effects of antibiotics. The development of antibiotic resistance is an ancient natural phenomenon firmly embedded in the common genome of microorganisms [17]. Acquired resistance can occur due to mutation of bacterial DNA or acquisition of resistance genes through horizontal gene transfer when DNA is transferred from one bacterium to another. Thus, wastewater treatment facilities become accumulators of bacteria resistant to antibiotics, and, due to the high diversity and density of the activated sludge microbes communities, serve as “hot spots” for horizontal transfer, recombination and spread of antibiotic resistance genes and antibiotic-resistant bacterial strains, which can then enter the environment [7,18].

Many recent works have testified a mechanism for maintaining antibiotic-resistant bacteria through co- or cross-resistance to trace elements or co-regulation of resistance pathways [19]. Thus, it appears likely that trace elements’ impact can directly select the metal-tolerant microorganisms simultaneously resistant to antibiotics. In our work, we investigated the resistance of metal-tolerant bacteria isolated from wastewater treatment plants and sewage sludge to various groups of antibiotics.

## 2. Results

### 2.1. Characteristics of the Studied Wastewater Treatment Facilities and Sewage Sludge

The environments in which the tested strains lived are characterized by the microelement composition indicated in Table 1. If the concentrations of trace elements in wastewater treatment facilities are quite low and comply with hygienic standards (with the exception of cadmium in the water of the secondary settling tank), then in sewage sludge the concentrations of these elements are significantly higher than in zonal soils. However, in accordance with national standards, only sludge older than 6 months is limited in agricultural use due to excessive concentrations of zinc and cadmium.

### 2.2. Antibiotic Resistance of Bacteria Isolated from Wastewater Treatment Facilities

The number of aerobic bacteria isolated from samples of water and activated sludge from treatment facilities was secondary settling tank active sludge—2.8 × 10^6^ cells/mL, secondary settling tank water—2.4 × 10^5^ cells/mL and digester—1.6 × 10^5^ cells/mL. Antibiotic resistance testing was carried out for 10 metal-tolerant strains [20] to 6 different antibiotics. The test results are shown in Table 2.

Cefepime is a fourth generation cephalosporin antibiotic (group of β-lactam antibiotics) with a bactericidal effect against most Gram-negative bacteria, including those producing β-lactamases [21]. Cefepime inhibits the synthesis of bacterial cell walls by covalently binding enzymes responsible for the final step in transpeptidation during peptidoglycan wall synthesis. This binding causes defects in the cell wall leading to autolysis and subsequent death of the organism [22]. Among the metal-tolerant strains of bacteria from wastewater treatment plants, strains of *Pseudomonas gessardii*, resistant to 5 mM Zn, were adapted to it. The production of β-lactamases capable of hydrolyzing the antibiotic is the most common mechanism of resistance exhibited by Gram-negative bacteria [23]. Cefepime resistance in *P. aeruginosa* is usually mediated by a combination of overproduction of chromosomal class C enzymes (*Amp*C) and/or upregulation of efflux pumps [24].

Ceftazidime belongs to the third-generation cephalosporins, a group of β-lactam antibiotics. The antibiotic is used to treat a wide range of infections such as urinary tract infections, hospital-acquired pneumonia, ventilator-associated infections and invasive and abdominal infections [25]. Strains of *Serratia proteamaculans* (5 mM Ni) and *Serratia proteamaculans* (5 mM Pb) at all antibiotic concentrations and *Pseudomonas gessardii* (3 mM Ni) at 20 μg/mg are resistant to it. Isolates of *Pseudomonas aeruginosa* that are resistant to cephalosporins are also frequently resistant to antibiotics of the carbapenem group (for example, meropenem) [26]. However, this is not typical for any metal-tolerant strains we isolated.

Meropenem belongs to a subgroup of carbapenems, the group of β-lactam antibiotics. It has a wide spectrum of action, causing bacteriostatic and bactericidal effects. Already at a concentration of 20 μg/mg, it suppresses all metal-tolerant strains. Antibiotics of the carbapenem group are the most effective bactericides for the treatment of infections caused by the most resistant microorganisms [27]. The resistance of some microorganisms to carbapenem can be gathered in three main groups. The first group includes mechanisms providing poor penetration of the antibiotic through the outer membrane of the bacterium or antibiotic efflux. The second are mechanisms that modify the toxicity target of the antibiotics through genetic mutations or post-translational modification of the target. The third includes mechanisms that act with enzyme-catalyzed modification and this is due to the production of beta-lactamases, which are able to inactivate carbapenems and so-called carbapenemases [27].

Streptomycin belongs to the first-generation aminoglycosides. Depending on the concentration, it can have both a bacteriostatic effect, causing disturbances in the translation process when interacting with the small subunit of ribosomes, and a bactericidal effect, damaging cell membranes [28]. It is quite effective against most identified metallotolerant strains. Only the strain of *Pseudomonas gessardii* (5 mM Zn), isolated from the sludge of the secondary settling tank, is resistant to it. The resistance of microorganisms to aminoglycosides arises through a variety of mechanisms. As a result of acquired mutations, the cell wall may be modified and the transport of antibiotics may be hampered. Resistance to these antibiotics may also increase due to the increased activity of efflux pumps. The most widespread mechanism of resistance to aminoglycosides is the inactivation of these antibiotics by aminoglycosides-modifying enzymes.

Kanamycin also belongs to the bactericidal aminoglycosides. *Pseudomonas* spp., *Streptococcus* spp. are insensitive or resistant to kanamycin. The strain of *Pseudomonas gessardii* (5 mM Zn) is resistant to this antibiotic at a concentration of 50 μg/mg and is also resistant to streptomycin.

Chloramphenicol has a bacteriostatic effect due to disruption of translation processes. It belongs to the group of amphenicols, dioxyaminophenylpropane derivatives. In our case, all metal-tolerant strains of the genus *Pseudomonas* showed resistance to this antibiotic. Resistance or decreased sensitivity to chloramphenicol is observed quite often in bacteria and is mediated by numerous mechanisms. The most common mechanism is the inactivation of chloramphenicol reactive groups by various types of enzymes, discussed in detail by Schwartz et al. [29]. It has been shown previously that O-phosporylation, hydrolytic degradation to p-nitrophenylserinol, and nitroreductation as inactivation pathways of chloramphenicol. But, it is obvious that the major enzymatic mechanism that inactivates chloramphenicol is acetylation by several types of specific acetyltransferases. The O-acetyl chloramphenicol derivatives have no properties of antibiotics because they do not bind to bacterial ribosomes. Other mechanisms may be associated with impaired transport of the antibiotic into the cell due to perturbations of the outer membrane or derivatization of chloramphenicol [30].

### 2.3. Antibiotic Resistance of Bacteria Isolated from Sewage Sludge

The number of aerobic colony-forming units in sewage sludge of different ages was 1.2 × 10^7^ cells/g in fresh (SS0), 3.1 × 10^7^ cells/g in 6-month-old (SS6), 3.8 × 10^6^ cells/g in 1-year-old (SS12) and 2.8 × 10^7^ cells/g in 5-year-old (SS60) sewage sludge.

**Figure 1:**
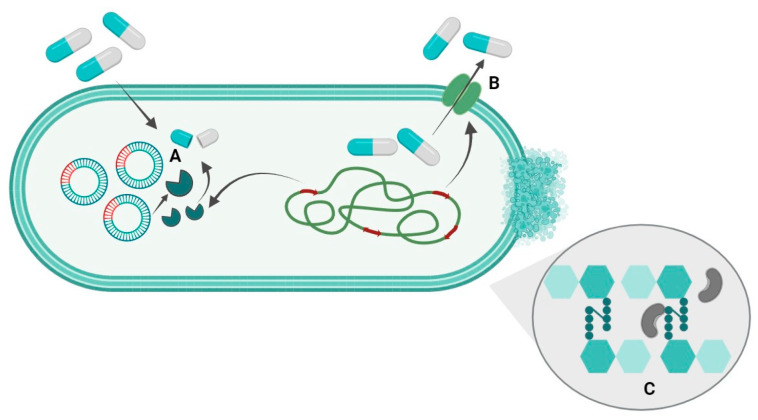
Major mechanisms of β-lactam resistance of bacteria (according to [31]). (**A**) Resistance by β-lactamase enzyme hydrolysis of the antibiotic; (**B**) resistance by active efflux of the antibiotic; (**C**) resistance by antibiotic-binding proteins modification and increased peptidoglycan synthesis.

The resistance of metal-tolerant bacteria to antibiotics belonging to the different subgroups of the group of β-lactam antibiotics is shown in Table 3. Major mechanisms of β-lactam resistance include resistance by β-lactamase enzyme hydrolysis of the antibiotic, resistance by active efflux of the antibiotic and resistance by antibiotic-binding protein modification and increased peptidoglycan synthesis [31] (Figure 1).

All metal-tolerant strains of bacteria from sewage sludge are resistant to ampicillin, a β-lactam semisynthetic antibiotic of the penicillin subgroup, at concentrations up to 100 μg/mg. Antibiotics of the same subgroup, penicillin and carbenicillin, were more effective—they suppressed the strains of *Rhodococcus*, as well as *Citrobacter freundii* (only carbenicillin).

*Stenotrophomonas maltophilia* SS0-5 and *Serratia liquefaciens* SS60-8 strains showed resistance to all studied antibiotics of the cephalosporin subgroup. The *Citrobacter freundii* strain SS60-12, on the contrary, was suppressed by all cephalosporins at any concentration. Obviously, metal-tolerant strains have the least resistance to ceftazidime—only 4 of 12 strains were resistant to it at maximum concentrations.

Bacteria are even less resistant to meropenem, a β-lactam antibiotic of the carbapenem subgroup. Only three strains (*Serratia fonticola* SS0-1, *Stenotrophomonas maltophilia* SS0-5, *Stenotrophomonas maltophilia* SS0-10) grew at maximum concentrations of this substance.

The resistance of the studied metal-tolerant strains to antibiotics of the tetracycline and aminoglycoside groups is shown in Table 4.

A group of tetracyclines are broad-spectrum bacteriostatic antibiotics that inhibit the synthesis of protein by binding the 30S ribosomal subunit and preventing the attachment of the aminoacyl-tRNA and as a result, interrupting the elongation phase of protein synthesis [32]. Metal-tolerant strains showed low resistance to tetracycline antibiotics (tetracycline and chlortetracycline). Only *Serratia fonticola* SS0-1 grew at all concentrations of these antibiotics and strains *Stenotrophomonas maltophilia* SS0-5, *Pseudomonas cedrina* SS60-7 and *Citrobacter freundii* SS60-12—at the lowest tetracycline concentration used (20 μg/mg). Three general tetracycline-specific resistance mechanisms have been described: efflux, ribosomal protection, and enzymatic inactivation of the antibiotic by bacterial cells [32].

Among metal-tolerant sewage sludge bacteria, *Stenotrophomonas maltophilia* strains showed resistance to all aminoglycoside antibiotics. Serratia fonticola SS0-1 was suppressed only by gentamicin at 20–40 and kanamycin at 40 μg/mg. The aminoglycoside antibiotic novobiocin, which is chemically an oxygen-containing heterocyclic compound, turned out to be the most ineffective among the aminoglycoside antibiotics against the bacteria studied. It suppressed the development of only *Rhodococcus qingshengii* strains.

Five strains are resistant to kanamycin at low concentrations (20 μg/mg): *Serratia fonticola* SS0-1, *Rhodococcus qingshengii* SS60-2, *Rhodococcus qingshengii* SS6-3, *Stenotrophomonas maltophilia* SS0-5, *Stenotrophomonas maltophilia* SS0-10 and at 40 μg/mg are resistant two strains of *Stenotrophomonas maltophilia* only. The remaining aminoglycosides quite effectively suppressed metal-tolerant strains of bacteria

Resistance of bacterial strains from sewage sludge to diaminopyrimidines, amphenicols and ansamycins is shown in Table 5.

Strains *Serratia fonticola* SS0-9 and *Serratia fonticola* SS12-11 were suppressed by minimal concentrations of trimethoprim (a group of diamidopyrimidines) and chloramphenicol (a group of amphenicols). Trimethoprim is a synthetic antibiotic that affects the synthesis of tetrahydrofolic acid, an important metabolite for amino acids and nucleotide synthesis. Chloramphenicol is a bacteriostatic antibiotic that binds to the 50S ribosomal subunit and inhibits the peptidyltransferase step in the synthesis of protein [33]. At the same time, strains of *Pseudomonas extremaustralis* SS0-6, *Pseudomonas cedrina* SS60-7 and *Serratia liquefaciens* (all resistant to 5 mmol Zn and 3 mmol Cu) were resistant to these antibiotics, even at maximum concentrations. A resistance of microbes to trimethoprim may be caused by changes in cell permeability, loss of bacterial antibiotic-binding capacity, and production specific or alterations of dihydrofolate reductase. The most important intrinsic mechanism is the synthesis of plasmid-mediated dihydrofolate reductases that have low affinity to trimethoprim. Resistance to chloramphenicol is mostly a result of the inactivation of the antibiotic by chloramphenicol acetyltransferase enzymes that acetylate the one [32].

From the groups of antibiotics presented in Table 6, the lowest resistance of metal-tolerant strains was to rifampicin (group of ansamycins) at a concentration of 100 μg/mg. The exception was the *Serratia fonticola* SS0-1 strain, which was resistant to most antibiotics of all groups studied. This strain was successfully suppressed only by maximum concentrations of ceftazidime (cephalosporin group) and trimethoprim (diamidopyrimidine group). The rifampicin (from the group of ansamycins) affects the β-subunit of the RNA polymerase, where it binds and inhibits the elongation of RNA shortly after initiation [34]. Rifampicin resistance arises due to single amino acid substitutions in the β-subunit of RNA polymerase [35,36].

## 3. Discussion

From all the metal-tolerant microorganisms isolated from wastewater treatment facilities in a large city, only the *Klebsiella pneumonia* strain (tolerant to 3 mM Cu) from the sludge of a secondary settling tank did not show resistance to the studied antibiotics at the concentrations considered. The remaining strains were mostly resistant to one antibiotic (*Serratia proteamaculans* (5 mM Ni), *Pseudomonas fragi* (3 mM Cd) from the sludge of secondary sedimentation tank, *Pseudomonas fragi* (3 mM Cd) from the water of secondary sedimentation tank, *Serratia proteamaculans* (5 mM Pb), *Pseudomonas fragi* (3 mM Pb) and *Pseudomonas brenneri* (3 mM Pb)). All strains of *Pseudomonas gessardii* showed resistance to 2 or more (up to 4) antibiotics at different concentrations.

Analysis of the resistance of metal-tolerant strains from sewage sludge to 18 antibiotics of various groups and subgroups shows that all of them exhibit resistance to antibiotics to varying degrees. A number of strains, for example, *Serratia fonticola* SS0-1, isolated from fresh sewage sludge and resistant to 5 mmol Cu and 3 mmol Pb, or *Stenotrophomonas maltophilia* SS0-5, also isolated from fresh sludge and resistant to 3 mmol Zn and 3 mmol Cu, are practically resistant to all antibiotics used at their various concentrations. Both strains are resistant to 14 antibiotics at their maximum concentrations and, additionally, to 3 and 2 antibiotics only at lower concentrations (respectively). At the same time, the *Rhodococcus qingshengii* strain SS60-2, isolated from 5-year-old sewage sludge and resistant to 5 mmol Co and 3 mmol Ni, Pb and Cu, as well as the *Rhodococcus qingshengii* strain SS6-3, isolated from 6-month-old sludge and resistant to 5 mmol Ni and 3 mmol Pb and Cu are resistant to 2 antibiotics at their maximum concentrations used and to another 2 at the minimum concentrations used only. The unrepresentativeness of the sample of strains does not allow us to draw a conclusion about the effect of sewage sludge age on antibiotic resistance, and this issue requires further study.

Our studies clearly show that most metal-tolerant microorganisms also have resistance to a wide range of antibiotics of various groups. Our results are confirmed by a significant amount of data from the literature [37,38,39,40,41,42]. Heavy metal co-resistance with antibiotics appears to be synergistic in bacterial isolates via similar mechanisms [39]. Martins et al. [37] showed that antibiotic resistance through the acquisition of plasmids can be induced by the selective pressure of heavy metals in the environment. In their studies, the strain of *Pseudomonas aeruginosa* EW32, isolated from an aquatic environment near industrial plants and a hospital, possessed a conjugative plasmid with core resistance to tetracycline and copper [37]. More than 70% of strains with multiple resistance to heavy metals also had multiple resistance to 11 antibiotics [43]. Most isolates isolated from the marine environment and showing tolerance to heavy metal (Cu, Zn, Hg, Pb, Cd) concentrations ranging from 12.5 to 6400 μg/mL were also resistant to 7 antibiotics [38]. Sajjad et al. [40] discovered by PCR amplification distinct antibiotic-resistant genes in bacteria from territories not subject to anthropogenic influence (glaciers). Among them, β-lactam genes *bla*_CTX-M_ (21.1–71.1%), *bla*_ACC_ (21.1–60.5%), tetracycline-resistant gene *tetA* (21.1–60.5%) and sulfonamide-resistant gene *sulI* (18.4–52.6%) dominated. Moreover, different metal-tolerant genes were reported in bacterial isolates, including mercury-resistant *merA* (21.1–63.2%), copper-resistant *copB* (18.4–57.9%), chromium-resistant *chrA* (15.8–44.7%) and arsenic-resistant *arsB* (10.5–44.7%). This highlights the co-selection and co-occurrence of metal-tolerant genes and antibiotic-resistant genes in remote glacier environments [40].

At the same time, there are very interesting experimental results that antibiotic resistance can be suppressed by the development of metalloid resistance mechanisms. Khaira et al. (2022) showed that *Delftia tsuruhatensis* demonstrated resistance to kanamycin but when grown in the presence of arsenic and kanamycin, bacteria lost resistance to the antibiotic. Therefore, it is suggested that the novel arsenate-resistant strain *Delftia tsuruhatensis* FK-01 has a unique ability to inhibit antimicrobial resistance [44].

Thus, the world of bacteria is so diverse that we can obviously find a wide variety of beneficial metabolic mechanisms in them [45]. However, it is clear that not all of them are widespread or have evolutionary significance. Our results and the overwhelming number of works by other authors presented in this paper confirm the dominant distribution of bacterial co-resistance to both antibiotics and metals/metalloids.

## 4. Materials and Methods

Samples of wastewater and active sludge from the secondary sedimentation tank and methane tank (digester) and different ages of mixed sewage sludge samples from the large city wastewater treatment plant were taken to isolate metal-tolerant microorganisms [20]. The concentration of heavy metals and metalloids in the waters and sludge of the treatment plant was measured by optical emission spectrometry with inductively coupled plasma (Optima-5300DV, Perkin Elmer, Waltham, MA, USA). The concentration of these elements in the collected samples of sewage sludge was measured by atomic absorption spectrometer with flame atomization of samples Analytik Jena contrAA^®^ 800 (Analytik, Jena, Germany). By cultivating on LB medium with salts Ni, Co, Cd, Zn, Pb, and Cu bacterial strains that were tolerant to these metals at concentrations of 3–5 mmol were selected and identified after Sanger sequencing [20]. Initial phylogenetic screening for the similarity of the 16S rRNA gene nucleotide sequences was carried out in the GenBank database (National Center for Biotechnology Information), using the BLAST software package. To finally determine the phylogenetic position of the strains, the subsequent 16S rRNA gene sequence was aligned with the corresponding sequences of the closest bacterial species using the CLUSTAL W program [46].

The identified HM-resistant strains were tested for resistance to antibiotics of different groups. Bacterial growth was assessed on LB agar medium of the following composition (g/L): NaCl (Helicon, Moscow, Russia)—5; yeast extract (Sigma-Aldrich, St. Louis, MO, USA)—5; tryptone (Sigma-Aldrich, St. Louis, MO, USA)—10; agar (Sigma-Aldrich, St. Louis, MO, USA)—15. After dissolving the components in distilled water, the medium was autoclaved for 30 min at a pressure of 0.5 atm. Antibiotics used for the experiment: penicillin, carbenicillin, gentamicin, chloramphenicol (USB, Douglasville, GA, USA); ampicillin, chlortetracycline, trimethoprim (Sigma-Aldrich, St. Louis, MO, USA); tetracycline, rifampicin (AppliChem GmbH, Darmstadt, Germany); kanamycin, streptomycin (JSC Biokhimik, Saransk, Russia); amikacin, ceftazidime (JSC Sintez, Kurgan, Russia); cefotaxime (Promomed, Moscow, Russia); ceftriaxone (JSC Borisov Medical Preparations Plant, Borisov, Belarus); novobiocin (HiMedia Lab, India); and meropenem (Belmedpreparaty, Minsk, Belarus), neomycin (NZYTech, Lisboa, Portugal). Stock solutions of antibiotics were prepared with an antibiotic concentration of 100 mg/mL. To do this, a sample of the substance (100 mg) was dissolved in 1 mL of sterile distilled water in a sterile Eppendorf tube. Then, a working solution with an antibiotic concentration of 20 mg/mL was prepared from the stock solution. The prepared antibiotic solution was added to a heated LB medium to the final concentrations indicated in Table 6. The antibiotics studied were used in two concentrations: strict and relaxed control. As a rule, these are 20 and 40 μg/mL of medium or 50 and 100 μg/mg of medium, depending on the generation of the antibiotic. Antibiotic concentrations were selected based on the European Committee on Antibiotic Susceptibility Testing EUCAST database [47], taking into account that the final concentration of the antibiotic in the medium should be higher than the minimum inhibitory concentration and ensure sustainable growth of resistant strains. Metal-tolerant strains of bacteria isolated from wastewater treatment facilities were tested for resistance to 6 antibiotics, and strains from wastewater sludge of different ages were tested for resistance to 18 different antibiotics.

Using the replica method, bacteria were transferred to an LB agar medium with various antibiotics. Petri dishes with bacteria and antibiotics were incubated at 28 °C for 48 h. Bacterial resistance was determined visually by the growth of fingerprint colonies on a medium with an antibiotic. Experiments were carried out in triplicate.

## 5. Conclusions

The results of our studies show the presence of cross-resistance of bacteria to both heavy metals (Pb, Cd, Zn, Cu, Ni, Co) and a wide range of antibiotics of various groups: β-lactam antibiotics, aminoglycosides, amphenicols, ansamycins, diaminopyrimidines and tetracyclines. Thus, it is possible for bacteria to develop resistance to antibiotics not only as a result of the use of antibiotics themselves, but also as a result of environmental pollution with heavy metals, and vice versa. Obviously, the mechanisms of bacterial adaptation to heavy metals are not specific, but are determined by genes also responsible for bacterial resistance to antibiotics. Both wastewater treatment facilities and sewage sludge are points of accumulation and spread of genes for resistance to heavy metals and antibiotics to other environmental objects. The mechanisms of such resistance are of undoubted interest and require detailed study.

## Figures and Tables

**Table 1:** The concentrations of heavy metals and metalloids (ppm) in samples from treatment facilities.

TE	Concentration of TE in Sewage Sludge of Different Ages							
	Sewage Sludge					Treatment Facilities		
	Fresh	1 Months	6 Months	1 Year	5 Years	Secondary Sedimentation Tank (Active Sludge)	Secondary Sedimentation Tank (Water)	Digester (Suspension)
Co	28.6	28.8	27.9	30.4	309.0	not determined		
Ni	66.8	33.3	72.3	50.1	69.0	0.0041	0.0089	0.1204
Cu	228.4	171.1	269.2	272.2	102.6	0.0037	0.0056	0.0248
Zn	1119.6	901.4	**1791.2**	**1865.8**	**2360.8**	0.00001	0.0002	0.0002
Cd	8.7	2.9	10.8	4.0	**33.0**	0.0037	**0.0053**	0.0186
Pb	38.0	27.1	37.4	27.6	60.0	0.0038	<0.0001	0.0050

Concentrations that exceed national standards for the relevant sites are highlighted in bold.

**Table 2:** Resistance of bacterial strains (black) from the treatment facilities objects to antibiotics.

Strain, Tolerance to the Metal(loid) and Origin	Antibiotic									
	Group of β-Lactam Antibiotics					Group of Aminoglycosides			Group of Amphenicols	
	Subgroup of Cephalosporins				Subgroup of Carbapenems					
	Cefepime		Ceftazidime		Meropenem	Streptomycin		Kanamycin	Chloramphenicol	
	μg/mg									
	20	40	20	40	20	50	100	50	50	100
*Serratia proteamaculans*, (5 mM Ni) from methane tank	−	−	+	+	−	−	−	−	−	−
*Pseudomonas gessardii*, (3 mM Ni) from sludge of secondary sedimentation tank	−	−	+	−	−	−	−	−	+	+
*Pseudomonas fragi*, (3 mM Cd) from sludge of secondary sedimentation tank	−	−	−	−	−	−	−	−	+	+
*Pseudomonas fragi*, (3 mM Cd) from water of secondary sedimentation tank	−	−	−	−	−	−	−	−	+	+
*Serratia proteamaculans*, (5 mM Pb) from sludge of secondary sedimentation tank	−	−	+	+	−	−	−	−	−	−
*Pseudomonas fragi*, (3 mM Pb) from water of secondary sedimentation tank	−	−	−	−	−	−	−	−	+	+
*Pseudomonas brenneri*, (3 mM Pb) from water of secondary sedimentation tank	−	−	−	−	−	−	−	−	+	+
*Pseudomonas gessardii*, (5 mM Zn) from sludge of secondary sedimentation tank	+	−	−	−	−	+	+	+	+	+
*Pseudomonas gessardii*. (5 mM Zn) from water of secondary sedimentation tank	+	+	+	−	−	−	−	−	+	+
*Klebsiella pneumonia*. (3 mM Cu) from sludge of secondary sedimentation tank	−	−	−	−	−	−	−	−	−	−

**Table 3:** Resistance of bacterial strains (black) from sewage sludge to β-lactam antibiotics.

Strain, Tolerance to the Metal(loid)	Subgroup of Penicillins						Subgroup of Cephalosporins						Subgroup of Carbapenems	
	Ampicillin		Penicillin		Carbenicillin		Cefotaxime		Ceftriaxone		Ceftazidime		Meropenem	
	μg/mg													
	50	100	50	100	50	100	20	40	20	40	20	40	20	40
*Serratia fonticola* SS0-1 (5 mmol Cu, 3 mmol Pb)	+	+	+	+	+	+	+	+	+	+	+	−	+	+
*Rhodococcus qingshengii* SS60-2 (5 mmol Co, 3 mmol Ni, Pb, Cu)	+	+	−	−	−	−	−	−	−	−	+	+	−	−
*Rhodococcus qingshengii* SS6-3 (5 mmol Ni, 3 mmol Pb, Cu)	+	+	−	−	−	−	−	−	−	−	+	+	−	−
*Pseudomonas fragi* SS0-4 (3 mmol Cd, Zn, Cu, Pb)	+	+	+	+	+	+	+	+	−	−	−	−	−	−
*Stenotrophomo-nas maltophilia* SS0-5 (3 mmol Zn 3 mmol Cu)	+	+	+	+	+	+	+	+	+	+	+	+	+	+
*Pseudomonas extremaustralis* SS0-6 (5 mmol Zn, 3 mmol Cu, Pb)	+	+	+	+	+	+	+	+	+	−	−	−	−	−
*Pseudomonas cedrina* SS60-7 (5 mmol Zn 3 mmol Cu)	+	+	+	+	+	+	+	+	+	+	−	−	−	−
*Serratia liquefaciens* SS60-8 (5 mmol Zn, 3 mmol Cu)	+	+	+	+	+	+	+	+	+	+	+	+	−	−
*Serratia fonticola* SS0-9 (5 mmol Pb, 3 mmol Ni)	+	+	+	+	+	+	+	+	+	+	−	−	−	−
*Stenotrophomonas maltophilia* SS0-10 (5 mmol Pb, 3 mmol Zn)	+	+	+	+	+	+	+	+	+	+	−	−	+	+
*Serratia fonticola* SS12-11 (5 mmol Pb, 3 mmol Cu)	+	+	+	+	+	+	+	+	+	+	−	−	−	−
*Citrobacter freundii* SS60-12 (5 mmol Pb, 3 mmol Zn)	+	+	+	+	−	−	−	−	−	−	−	−	−	−

**Table 4:** Resistance of bacterial strains (black) from sewage sludge to tetracyclines and aminoglycosides.

Strain, Tolerance to Metal(loids)	Antibiotics															
	Group of Tetracyclines				Group of Aminoglycosides											
	Tetracycline		Chlortetracycline		Streptomycin		Amikacin		Gentamicin		Neomycin		Novobiocin		Kanamycin	
	μg/mg															
	20	40	20	40	50	100	20	40	20	40	20	40	20	40	20	40
*Serratia fonticola* SS0-1 5 mmol Cu, 3 mmol Pb	+	+	+	+	+	+	+	+	−	−	+	+	+	+	+	−
*Rhodococcus qingshengii* SS60-2 5 mmol Co, 3 mmol Ni, Pb, Cu	−	−	−	−	−	−	−	−	−	−	−	−	−	−	+	−
*Rhodococcus qingshengii* SS6-3 5 mmol Ni, 3 mmol Pb, Cu	−	−	−	−	−	−	−	−	−	−	−	−	−	−	+	−
*Pseudomonas fragi* SS0-4 3 mmol Cd, Zn, Cu, Pb	−	−	−	−	−	−	−	−	−	−	−	−	+	+	−	−
*Stenotrophomonas maltophilia* SS0-5 3 mmol Zn, 3 mmol Cu	+	−	−	−	+	+	+	+	+	+	+	+	+	+	+	+
*Pseudomonas extremaustralis* SS0-6 5 mmol Zn, 3 mmol Cu, Pb	−	−	−	−	−	−	−	−	−	−	−	−	+	+	−	−
*Pseudomonas cedrina* SS60-7 5 mmol Zn, 3 mmol Cu	+	−	−	−	−	−	−	−	−	−	−	−	+	+	−	−
*Serratia liquefaciens* SS60-8 5 mmol Zn, 3 mmol Cu	+	−	−	−	−	−	−	−	−	−	−	−	+	+	−	−
*Serratia fonticola* SS0-9 5 mmol Pb, 3 mmol Ni	−	−	−	−	−	−	−	−	−	−	−	−	+	+	−	−
*Stenotrophomonas maltophilia* SS0-10 5 mmol Pb, 3 mmol Zn	−	−	−	−	+	+	+	+	+	+	+	+	+	+	+	+
*Serratia fonticola* SS12-11 5 mmol Pb, 3 mmol Cu	−	−	−	−	−	−	−	−	−	−	−	−	+	+	−	−
*Citrobacter freundii* SS60-12 5 mmol Pb, 3 mmol Zn	+	−	−	−	−	−	−	−	−	−	−	−	+	+	−	−

**Table 5:** Resistance of bacterial strains (black) from sewage sludge to tetracyclines and aminoglycosides. Cephalosporins, carbopenems, amphenicols, ansamycins and diaminopyrimidines.

Strain, Tolerance to Metal(loids)	Antibiotics					
	Group of Diaminopyrimidines		Group of Amphenicols		Group of Ansamycins	
	Trimethoprim		Chloramphenicol		Rifampicin	
	μg/mg					
	20	40	25	50	50	100
*Serratia fonticola* SS0-1 5 mmol Cu, 3 mmol Pb	+	−	+	+	+	+
*Rhodococcus qingshengii* SS60-2 5 mmol Co, 3 mmol Ni, Pb, Cu	+	−	−	−	−	−
*Rhodococcus qingshengii* SS6-3 5 mmol Ni, 3 mmol Pb, Cu	+	−	−	−	−	−
*Pseudomonas fragi* SS0-4 3 mmol Cd, Zn, Cu, Pb	+	−	+	+	−	−
*Stenotrophomonas maltophilia* SS0-5 3 mmol Zn, 3 mmol Cu	+	+	−	−	+	−
*Pseudomonas extremaustralis* SS0-6 5 mmol Zn, 3 mmol Cu, Pb	+	+	+	+	−	−
*Pseudomonas cedrina* SS60-7 5 mmol Zn, 3 mmol Cu	+	+	+	+	−	−
*Serratia liquefaciens* SS60-8 5 mmol Zn, 3 mmol Cu	+	+	+	+	+	−
*Serratia fonticola* SS0-9 5 mmol Pb, 3 mmol Ni	−	−	−	−	−	−
*Stenotrophomonas maltophilia* SS0-10 5 mmol Pb, 3 mmol Zn	+	−	−	−	−	−
*Serratia fonticola* SS12-11 5 mmol Pb, 3 mmol Cu	−	−	−	−	+	−
*Citrobacter freundii* SS60-12 5 mmol Pb, 3 mmol Zn	+	−	+	−	+	−

**Table 6:** Antibiotic concentrations used for antibiotic resistance study.

Antibiotic	Concentration in the LB Medium µg/mL	Antibiotic	Concentration in the LB Medium µg/mL
Amikacin	20, 40	Kanamycin	20, 40
Ampicillin	50, 100	Meropenem	20, 40
Gentamicin	20, 40	Neomycin	20, 40
Carbenicillin	20, 40	Novobiocin	20, 40
Cefepime	20,40	Penicillin	50, 100
Cefotaxime	20, 40	Rifampicin	50, 100
Ceftazidime	20, 40	Streptomycin	50, 100
Ceftriaxone	20, 40	Tetracycline	20, 40
Chloramphenicol	25, 50	Trimethoprim	20, 40

## Data Availability

Data are contained within the article.
